# Increasing Pyruvate Concentration Enhances Conidial Thermotolerance in the Entomopathogenic Fungus *Metarhizium robertsii*

**DOI:** 10.3389/fmicb.2019.00519

**Published:** 2019-03-20

**Authors:** Congcong Wu, Xing Zhang, Weiguo Fang

**Affiliations:** MOE Key Laboratory of Biosystems Homeostasis and Protection, Institute of Microbiology, Zhejiang University, Hangzhou, China

**Keywords:** *Metarhizium*, heat stress, pyruvate accumulation, biological control, entomopathogenic fungi

## Abstract

The fungal entomopathogens *Metarhizium* spp. have been developed as environmentally friendly mycoinsecticides. However, heat stress severely reduces the viability of *Metarhizium* conidia in the field, which is an important obstacle to the successful use of these mycoinsecticides. Heat treatment induces rapid accumulation of pyruvate, which timely scavenges heat-induced ROS (reactive oxygen species) in hyphal cells of *M. robertsii*. However, in heat-treated conidia, pyruvate accumulation occurs later than the rapid production of ROSs, which could harm the conidial cells. In the present study, a transgenic *M. robertsii* strain was constructed with the pyruvate kinases gene overexpressed during conidiation. Two independent transformants of the transgenic strain produced conidia under optimal conditions with elevated pyruvate concentration. This inhibits the rapid heat-induced ROS production and prevents the collapse of mitochondrial membrane potential, thereby increasing conidial tolerance to heat stress. In conclusion, the tolerance of *M. robertsii* conidia to heat stress was improved by increasing the conidial pyruvate concentration, which could be translated into a more effective pest control.

## Introduction

The entomopathogenic fungi *Metarhizium* spp. are being developed as environmentally friendly mycoinsecticides (Li et al., 2010). The conidium is the propagule that initiates pathogenesis, and it is thus the acting component in the mycoinsecticides. In spite of *Metarhizium* being adaptable to many environments, abiotic stresses, such as heat and UV radiation, severely reduce the viability of conidia in the field, and these stressors are important obstacles to the successful use of *Metarhizium* spp. (Fang et al., 2012; Liao et al., 2014; Zhao et al., 2016). Conidial tolerance to abiotic stresses can be improved by selecting the optimal growth substrate and conditions for conidial production (Rangel et al., 2015). Based on the molecular mechanisms of abiotic stress tolerance, conidial tolerance to abiotic stresses can also be achieved by genetic engineering. Reactive oxygen species (ROSs) are upregulated by multiple stresses, and ROS scavengers reduce the level of stress-induced ROSs and are thus involved in stress tolerance. Overexpressing genes that encode a SOD (superoxide dismutase) and a bacterial thioredoxin increases heat tolerance in the entomopathogenic fungus *Beauveria bassiana* (Xie et al., 2010; Ying and Feng, 2011). The expression of the small heat shock protein HSP25 is upregulated when *M. robertsii* is grown at extreme temperatures or in the presence of oxidative or osmotic agents. Overexpressing HSP25 increases the growth of *M. robertsii* under heat stress and enhances the tolerance of heat shock-treated conidia to osmotic stress (Liao et al., 2014). It is likely that the discovery of other mechanisms of heat stress tolerance would provide more approaches for the further improvement of mycoinsecticides.

Recently, it was found that pyruvate is a fungal ROS scavenger. In heat treated hyphae, pyruvate accumulation is coincident with ROS production, and pyruvate timely scavenges heat-induced ROSs, thereby reducing the damage caused by ROSs to cellular proteins and the mitochondrial membrane potential. Heat treatment also upregulates the production of ROSs and pyruvate in the conidia, but pyruvate accumulation occurs later than ROS burst, and the ROS level thus remains high until pyruvate accumulation (Zhang et al., 2017). As pyruvate accumulation functions as the first line of defense against heat-induced ROSs in conidia (Zhang et al., 2017), it is likely that the ROSs cause damages to the conidia before pyruvate accumulation. In present study, we increased the pyruvate concentration in unstressed conidia by overexpressing a pyruvate kinase gene. This reduced the level of heat-induced ROSs and consequently improved conidial tolerance to heat stress.

## Results

### Increasing Pyruvate Concentration in the Conidia by Overexpressing a Pyruvate Kinase Gene

In our previous study, we found that heat treatment induced pyruvate accumulation in *M. robertsii* by upregulating the pyruvate kinase gene (MAA_06851) for pyruvate production and by downregulating many genes involved in pyruvate consumption, including MAA_02871 and MAA_08787 (Zhang et al., 2017). The pyruvate kinase (MAA_06851) catalyzes the transfer of a phosphate group from phosphoenolpyruvate to adenosine diphosphate, yielding pyruvate and ATP. MAA_02871 encodes a pyruvate transporter that moves pyruvate from the cytoplasm into mitochondria for the TCA cycle, while MAA_08787 encodes the β-subunit of the pyruvate dehydrogenase E1 component. To increase the pyruvate concentration in the conidia that were grown under the optimal conditions [on potato dextrose agar (PDA) at 26°C], we overexpressed the pyruvate kinase gene (MAA_06851) or knocked down using the antisense RNA method the two genes (MAA_02871 and MAA_08787) that are involved in pyruvate consumption. Pyruvate is located at a key intersection in the network of metabolic pathways, so constitutive modification of pyruvate metabolic pathway could harm some fungal development stages. Meanwhile, as described above, conidium is the acting component in mycoinsecticides, and so we intended to increase pyruvate concentration only in conidia to overcome the time difference between the heat-induced ROS production and pyruvate accumulation and enhance conidial heat tolerance. To do this, the three genes described above were controlled by the conidiation specific promoter of the polyketide synthase gene *Pks1*, which is only highly expressed during conidiation and involved in synthesis of conidial pigments (Zeng et al., 2017, 2018).

**Figure 1:**
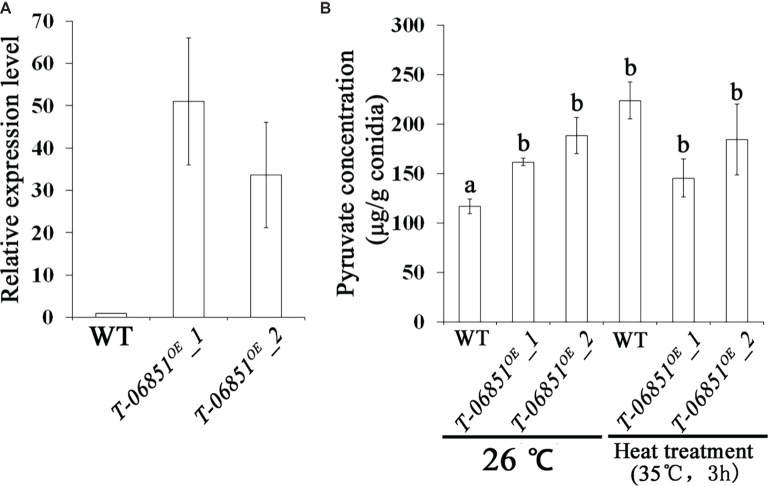
Increasing pyruvate concentration in *M. robertsii* conidia by overexpressing the pyruvate kinase gene *MAA_06851*. **(A)** qRT-PCR confirmation of the overexpression of *MAA_06851* in the conidia of two transformants (*T-06851^OE^-1* and *T-06851^OE^-2*). The gene expression levels during conidiation in the two transformants (5 days after inoculation of conidia on PDA) were calculated relative to that of the wild-type (WT) strain, which was set to 1. **(B)** The pyruvate concentration in the conidia grown at optimal conditions (26°C on PDA for 14 days), and in the heat-treated conidia. Data are expressed as mean ± standard error (SE). Values with different letters are significantly different ( *p* < 0.05, Tukey’s test in one-way ANOVA). All assays were repeated three times with three replicates per repeat.

For the two genes involved in pyruvate consumption, no significant differences in the expression levels during conidiation were observed between the wild-type (WT) strain and the five randomly selected transformants (*p* > 0.05, Tukey’s test in one-way ANOVA), indicating failure of gene expression suppression using the antisense RNA method. However, we obtained the transgenic strain *T-MAA_06851^OE^* with the pyruvate kinase gene (*MAA_06851*) overexpressed in the conidiating mycelium that was grown PDA for 5 days at optimal temperature (26°C) (Figure 1A). Two independent transformants (*T-MAA_06851^OE^*-1 and *T-MAA_06851^OE^*-2) were subjected to the pyruvate assay. Grown under the optimal conditions (at 26°C on PDA for 14 days), the pyruvate concentrations in the conidia of the two transformants were significantly higher than the WT strain (*p* < 0.05, Tukey’s test in the one-way ANOVA). No significant difference was found between these two transformants (*p* > 0.05, one-way ANOVA) (Figure 1B). The pyruvate concentrations in the conidia of the transformants grown under the optimal conditions were comparable to the WT conidia that had been heat treated (*p* > 0.05, Tukey’s test in the one-way ANOVA). However, heat treatment did not further increase the pyruvate concentration in the conidia of the transformants (*p* > 0.05, Tukey’s test in the one-way ANOVA) (Figure 1B).

### Increasing the Pyruvate Concentration Reduces the Level of Total Ross and Prevents the Heat-Induced Collapse of Mitochondrial Membrane Potential in Conidia

**Figure 2:**
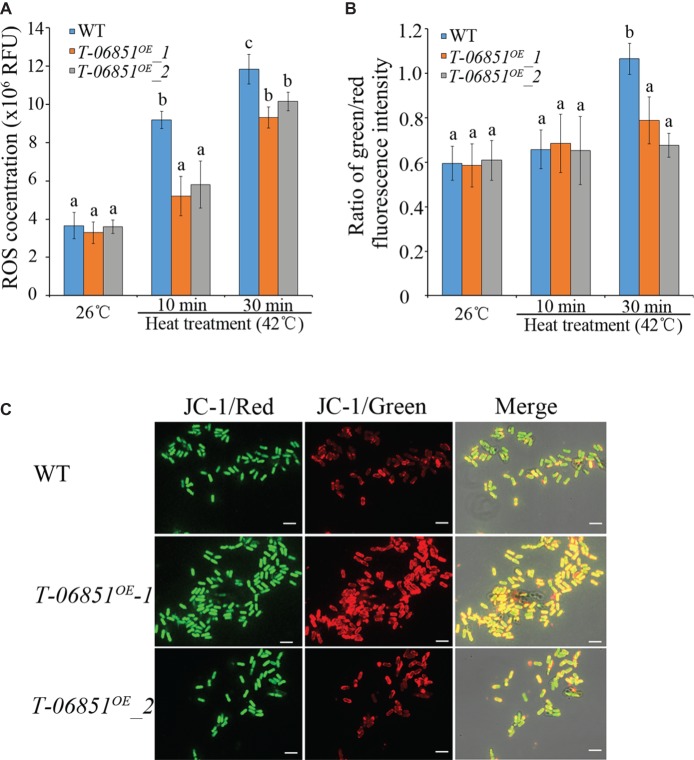
Increasing pyruvate concentration reduces the ROS level and prevents the collapse of mitochondrial membrane in conidia treated with heat (42°C, a widely used temperature for assaying heat stress tolerance of *M. robertsii* conidia). **(A)** ROS levels in the conidia at the optimal temperature (26°C) and the heat-treated conidia. RFU: relative fluorescence units. **(B)** Pyruvate prevents the collapse of mitochondrial membrane potential. The green/red fluorescence intensity ratio represents the extent to which the mitochondrial membrane potential collapses. **(C)** Typical pictures representative of five repeats of JC-1 staining for analysis of the mitochondrial membrane potential collapses. Bars, 10 μm. Values with different letters are significantly different (Tukey’s test in one-way ANOVA, *p* < 0.05). WT: The wild-type strain; *T-06851^OE^-1* and *T-06851^OE^-2*: two independent transformants with elevated pyruvate concentrations in the conidia.

We then investigated whether elevating pyruvate concentration could reduce the ROS level in heat-treated conidia. At the optimal temperature (26°C), no significant difference in the ROS level was found between WT and the two transformants (*T-MAA_06851^OE^*-1 and *T-MAA_06851^OE^*-2) (*p* > 0.05, Tukey’s test in one-way ANOVA). In previous studies (e.g., Fang et al., 2010; Rangel et al., 2015), 42°C was often used to assay heat tolerance of *Metarhizium* conidia in context of development of mycoinsecticides. In this study, we thus used 42°C to assay the heat tolerance of the transformants. The heat treatment (42°C for 10 min) significantly increased the ROS level in WT conidia (*p* < 0.05, Tukey’s test in one-way ANOVA), but this did not affect the level of ROSs in the two transformants (*p* > 0.05, Tukey’s test in one-way ANOVA) (Figure 2A). The ROS level in the heat-treated WT conidia was thereby significantly higher than the two transformants, which were not significantly different from each other. The elongated heat treatment (42°C for 30 min) also significantly increased the ROS level in the two transformants, which were still significantly lower than the WT strain (*p* < 0.05, Tukey’s test in one-way ANOVA) (Figure 2A).

ROSs destabilizes the mitochondrial membrane potential (Wang et al., 2007). At the optimal temperature (26°C), the ratio of green/red fluorescence intensity in the WT conidia was not significantly different from the two transformants (*T-MAA_06851^OE^*-1 and *T-MAA_06851^OE^*-2; Figures 2B,C), indicating that they had the same mitochondrial membrane potential. Heat treatment (42°C for 10 min) had no significant impact on mitochondrial membrane potential in both the WT strain and the transformants (Figure 2B). However, after heat treatment at 42°C for 30 min, the ratio of green/red fluorescence intensity in the WT conidia was significantly greater than the transformants (*p* < 0.05, Tukey’s test in one-way ANOVA) (Figures 2B, C); the ratio of green/red fluorescence intensity in the heat treated transformants was not different from those grown under the optimal conditions, indicating that the heat treatment induced a significant mitochondrial membrane potential collapse in WT but had no significant impact on the transformants.

### Increasing the Pyruvate Concentration Improved Conidial Tolerance to Heat Stress

The impact of the elevated production of conidial pyruvate on abiotic stress tolerance was further assayed. Under optimal conditions [grown in the nutrient-rich medium 1/2 SDY (Sabouraud dextrose broth supplemented with 1% yeast extract) at 26°C], the GT_50_ [time (hours) required for 50% of conidia to germinate] value of the WT strain was not significantly different from the two transformants (*T-MAA_06851^OE^*-1 and *T-MAA_06851^OE^*-2) (*p* > 0.05, one-way ANOVA), and the transformants were not significantly different from each other (*p* > 0.05, one-way ANOVA). The relative germination inhibition (defined in the Materials and Methods section) was used to compare the conidial germination of the WT strain and the transformants under several abiotic stresses. No significant differences in tolerance to UV radiation, osmotic stress, and oxidative stress were found between the WT strain and the two transformants (*p* > 0.05, one-way ANOVA; Table 1). However, the conidia of the two transformants treated with heat at 40°C for 60 min or at 42°C for 30 min germinated significantly faster than that of the heat-treated WT strain (Table 1), but at 36 h, after the heat treated conidia were incubated at the optimal temperature (26°C), nearly all conidia germinated.

**Table 1:** Relative germination inhibition of the WT strain and two transformants with elevated pyruvate concentration in the conidia under the four abiotic stresses.

	UV radiation	Hyperosmotic stress	Oxidative stress	Heat stress	
				40°C (60 min)	42°C (30 min)
WT	0.22 ± 0.02^a^	0.30 ± 0.06^a^	0.04 ± 0.01^a^	0.23 ± 0.009^a^	0.60 ± 0.04^a^
*T-06851^OE^-1*	0.14 ± 0.02^a^	0.33 ± 0.07^a^	0.03 ± 0.01^a^	0.12 ± 0.013^b^	0.45 ± 0.03^b^
*T-06851^OE^-2*	0.18 ± 0.03^a^	0.31 ± 0.04^a^	0.05 ± 0.01^a^	0.14 ± 0.006^b^	0.46 ± 0.02^b^

Within the same abiotic stress treatment, the values followed by different letters were significantly different (*p* < 0.05, Tukey’s test in a one-way ANOVA). All assays were repeated three times with three replicates per repeat.

The numerical values in the table: the relative germination inhibition of a given stressor on each strain was calculated as (Gc-Gt)/Gc, where Gc and Gt denote the GT_50_ [time (hours) taken for 50% of conidia to germinate] of the stressed and unstressed conidia, respectively.

The pathogenicity was assayed on *Drosophila melanogaster* adult flies. Inoculations were conducted by topically applying conidia onto the insect cuticle. No significant difference in virulence was found among the WT strain [LT_50_ (time taken to kill 50% of insects) = 12.1 ± 0.21 days] and the two transformants (*T-MAA_06851^OE^*-1: LT_50_ = 12.3 ± 0.35 days; *T-MAA_06851^OE^*-2: LT_50_ = 12.6 ± 0.41 days; *p* > 0.05, Tukey’s test in one-way ANOVA).

### Discussion

Heat treatment induces a rapid ROS burst in the conidia of *M. robertsii*, and the ROS level remains high until the ROS scavenger pyruvate is upregulated (Zhang et al., 2017). In the present study, we constructed two transformants that produce conidia under optimal conditions with elevated pyruvate concentration. This inhibited the rapid ROS burst induced by heat and prevented the collapse of the mitochondrial membrane potential, thereby increasing conidial tolerance to heat stress.

Fungal cells exposed to a mild stress develop tolerance not only to higher doses of the same stress but also to other stresses (Hohmann and Mager, 2003). This phenomenon is called cross protection. In the hyphae of *M. robertsii*, pyruvate accumulation contributes to the cross-protection against multiple abiotic stresses, including heat, oxidative, osmotic/salt stresses, and UV radiation, which are largely attributed to these four stressors inducing the accumulation of pyruvate that scavenges stress-induced ROSs (Zhang et al., 2018). However, in the present study, we found that increasing the pyruvate concentration did not improve conidial tolerance to oxidative and osmotic/salt stresses and UV radiation. This discrepancy could have resulted from the difference in metabolic states or mechanisms for abiotic stress tolerance between the hyphae and conidia.

Although the expression of the pyruvate kinase gene was increased over 30-fold in the two analyzed transformants, the pyruvate concentration only increased by approximately 1.5-fold. This is similar to the stress-induced pyruvate accumulation in hyphae. Pyruvate has metabolic and redox properties and is located at a key intersection in the network of metabolic pathways (Roudier and Perrin, 2009). The fold of increase (1.5-fold) in pyruvate caused by stresses and by the genetic modification of pyruvate metabolism could be the greatest extent that fungal cells can resist. Changes in pyruvate concentration, to a greater extent, could otherwise be lethal or impair the fungal growth (Biagini et al., 2001; Zhang et al., 2017).

In conclusion, increasing the conidial pyruvate concentration by overexpressing a pyruvate kinase gene improved the tolerance of the conidia of *M. robertsii* to heat stress.

### Materials and Methods

#### Construction of Transgenic Strains With Increased Pyruvate Concentration in Conidia

In order to construct antisense RNA plasmids to knock down two genes (MAA_02871and MAA_08787) involved in pyruvate consumption during the conidiation of *M. robertsii*, the promoter region (645 bp) of the *Pks1* gene (MAA_07745) (Zeng et al., 2018) was used. Approximately 200 bp of the DNA fragment corresponding to part of the coding sequence of a target gene was amplified by PCR using the high-fidelity Taq DNA polymerase (Toyobo, Japan) and inserted downstream of the promoter of the *Pks1* gene in the plasmid pPK2-bar-GFP-Pro (Zeng et al., 2018) to produce the antisense RNA plasmids, which were subsequently transformed into WT *Metarhizium robertsii* mediated by *Agrobacterium tumefaciens* AGL-1 as described (Xu et al., 2014). *M. robertsii* ARSEF2575 was obtained from the Agricultural Research Service Collection of Entomopathogenic Fungi. The fungal transformants were selected based on their resistance to the herbicide glufosinate ammonium (Sigma-Aldrich, USA), which was achieved by the *bar* gene in the plasmids (Xu et al., 2014). The fungal transformants with herbicide resistance were grown on PDA plates at 26°C for 5 days, and the conidiating mycelium was collected for RNA extraction and quantitative RT-PCR analyses (described below) to screen for transformants with expression of target genes reduced. All PCR products were confirmed by sequencing. The primers used in the present study are summarized in Table 2.

**Table 2:** Primers used in this study.

Primers	Sequences	Note
P7745-5 P7745-3	GGCCCGGGAGGGCACTCAAATCATA GGGATATCGTTGATCCGAAGGTTGC	Cloning the promoter of MAA_07745
6851-ORF5 6851-ORF3	GGTTTAAAATGGCTGCTGCTCAACA GGTTTAAACTAAGCCTGGCCAATTC	Cloning the genomic region of ORF of MAA_06851
2781-RNAi5 2781-RNAi3	GGGATATCTTCTGCAGCCAGTCGGT GGGATATCCATGAAGATGCAAATGA	Cloning part of MAA_02781’s ORF
8787-RNAi5 8787-RNAi3	GGGATATCTCTTCTCAAGGCTGCTA GGGATATCATGGTCAGGGCAAGAAT	Cloning part of MAA_08787’s ORF
6851-qRT5 6851-qRT3	GGCAATGCTATCACTGACGG CAATGATACCACCAGCAGCC	qRT-PCR analysis of MAA_06851
2781-qRT5 2781-qRT3	CCCTCAAACCACCCATGTTG GTGATGCCCCATGTGATGTC	qRT-PCR analysis of MAA_02781
8787-qRT5 8787-qRT3	ACCACTCCTGTCTTCTTCGG GTAGGCACCATTGTACTGCG	qRT-PCR analysis of MAA_08787
Act5 Act3	TCCTGACGGTCAGGTCATC CACCAGACATGACGATGTTG	Reference gene for qRT-PCR analysis
Tef5 Tef3	AGGCTGACTGCGCTATTCTC ACTTGGTGGTGTCCATCTTG	Reference gene for qRT-PCR analysis

In order to construct the plasmid overexpressing the pyruvate kinase gene (MAA_06851), its coding sequence was cloned by PCR using the high-fidelity Taq DNA polymerase (Toyobo, Japan) and inserted downstream of the *Pks1*’s promoter in the plasmid pPK2-bar-GFP-Pro (Zeng et al., 2018). The resulting plasmid pPK2-bar-GFP-P7745–06851 was subsequently transformed into the WT *M. robertsii* mediated by *A. tumefaciens*. The expression of MAA_06851 during conidiation on PDA plates was assayed as described for MAA_02871 and MAA_08787.

#### RNA Preparation and Quantitative RT-PCR Analysis

TRIzol reagent (Life Technologies, USA) was used to extract total RNA from conidia collected from the conidiating mycelium that was grown at 26°C on PDA plates for 5 days. Two genes (*act* and *tef*) were used as internal standards in the quantitative RT-PCR analyses (Fang and Bidochka, 2006). The relative normalized transcript level of a gene was computed using the 2^−ΔΔCt^ method (Livak and Schmittgen, 2001). cDNA was synthesized using a ReverTra Ace qPCR RT Master Mix with a gDNA remover (Toyobo, Japan). The quantitative PCR was conducted using a Thunderbird SYBR qPCR Mix (no ROX) (Toyobo, Japan). These experiments were repeated three times with three replicates per repeat.

#### Quantification of Pyruvate and Total Ross in Conidia

Determination of pyruvate and ROS concentrations in conidia was conducted as previously described (Zhang et al., 2017). Briefly, approximately 10^8^ conidial cells were treated with liquid nitrogen for 5 min to kill the conidia. The inactivated conidia were incubated at 26°C for 2 h in 0.01% Zygolase (Seikagaku Biobusiness, Japan) and 10% snailase (Solarbio, China) to partially remove the cell walls. Then, the treated conidia were washed twice with phosphate-buffered saline (PBS) buffer, suspended in 400 μl of PBS, and homogenized (10 min at 65 Hz) with ceramic beads (diameter = 1 mm) in a grinder (Jinxing, Shanghai China). Afterward, the supernatant was subjected to pyruvate quantification using the Pyruvate Assay Kit (Abcam, USA). For quantification of total ROSs, the supernatant was diluted 500-fold with the PBS buffer, and 50 μl of the diluted supernatant was subjected to ROS quantification using the Oxiselect *in vitro* ROS/RNS Assay Kit (Cell Biolabs, Inc., USA).

To prepare heat-treated conidia, 14 days old fungal cultures on PDA grown at 26°C were incubated at higher temperatures for different amounts of time, and conidia were then collected for pyruvate and ROS assays. As previously described (Zhang et al., 2017), the conidia for pyruvate assays were treated at 35°C. For ROS assays, 42°C was used for heat treatment.

All quantification assays were repeated three times with two replicates per repeat.

#### Assays of Mitochondrial Membrane Potential

The mitochondrial membrane potential of conidia was assayed with the JC-1 Staining Kit (Beyotime Biotechnology, Shanghai, China). Briefly, conidia were stained in the JG-1 (a mitochondrial membrane potential-dependent dye) staining solution for 30 min, and then the conidia were rinsed three times with the staining buffer. The fluorescence intensities at wavelengths of 490 (excitation) and 530 (emission) nm and at 525 (excitation) and 590 (emission) nm were measured by a confocal microscopy (Zeiss, Germany), and their ratio was calculated with the ImageJ software (NCBI). The experiments were repeated five times with three replicates per repeat.

#### Assays of Tolerance to Abiotic Stresses

Assays of conidial tolerance to UV radiation, oxidative stress, hyperosmotic stress, and heat stress were conducted as previously described (Zeng et al., 2017, 2018). Conidia were collected from the fungal cultures that were grown at 26°C on PDA plates for 14 days. For the assay of tolerance to UV radiation, the conidia were exposed to a 312-nm (280–320 nm) UV-B wavelength at 0.2 J cm^−2^ in a Bio-Sun++ chamber (VilberLourmat, Marne-la-Vallée, France). Then, the irradiated conidia were incubated at 26°C, and the conidial germination was observed every 2 h using an inverted microscope (Leica, Germany).

For hyperosmotic and oxidative stress, tolerance was assayed by measuring the germination rate of the conidia in 1/2 SDY supplemented with 0.75 M of KCl or 3 mM of H_2_O_2_, respectively.

The tolerance of conidia to heat stress was investigated by incubating the conidial suspensions at 40 or 42°C for a certain period of time (10, 30, and 60 min), and transferring to 26°C to continue growth. The germination was checked every 2 h. The optimal temperature (26°C) was used as a control.

The relative germination inhibition of a given stressor on each strain was calculated as (Gc-Gt)/Gc (Wang et al., 2014), where Gc and Gt denote the GT_50_ [time (hours) required for 50% conidia to germinate] of the stressed and unstressed conidia, respectively. All assays were repeated three times with three replicates per repeat.

#### Pathogenicity Assays

Pathogenicity assays were conducted as described (Wang et al., 2017). Inoculations were performed by immersing flies in conidial suspensions followed by vortexing gently for 10 s. *D. melanogaster* (*w^1118^* stock number 5905) was maintained at 27°C, 85% humidity, on food made without Tegosept and propionic acid. The recipe for fly food can be found at http://cshprotocols.cshlp.org/content/2014/9/pdb.rec081414.full?text_only=true. We bioassayed three tubes of 30 adult flies (aged 2–4 days) with a conidial suspension (2.5 × 10^4^ conidia/ml of water) produced from 14 days old plates. Control flies were treated with water alone. Mortality was recorded every 12 h. All bioassays were repeated three times. LT_50_ values were calculated using the *SPSS* program (Chicago, USA).

## Author Contributions

CW and XZ performed the experiment. XZ and WF conceived the idea of the study. WF wrote the manuscript.

### Conflict of Interest Statement

The authors declare that the research was conducted in the absence of any commercial or financial relationships that could be construed as a potential conflict of interest.
